# Ruptured hepatic artery aneurysm presenting as abdominal pain: a case report

**DOI:** 10.4076/1757-1626-2-8529

**Published:** 2009-09-08

**Authors:** Chang-Bon Man, Kasim A Behranwala, Malcolm S Lennox

**Affiliations:** Department Of General Surgery, Queen Elizabeth II HospitalHowlands, Welwyn Garden City, AL7 4HQUK

## Abstract

Aneurysms of the hepatic artery are rare. This patient presented to the emergency department with severe epigastric pain and subsequently became haemodynamically unstable. Plain abdominal radiograph showed a ring lesion in the right upper quadrant, ultrasound scan demonstrated a mass with arterial blood flow, and computed tomography revealed a left hepatic artery aneurysm. At surgery, the ruptured aneurysm was identified and the left hepatic artery was successfully ligated. Prompt diagnosis is of paramount importance and crucial information may be gleamed from investigations in the emergency department. If a ruptured aneurysm is diagnosed, we recommend prompt referral to a surgical team for definitive management.

## Case presentation

**Figure 1.**
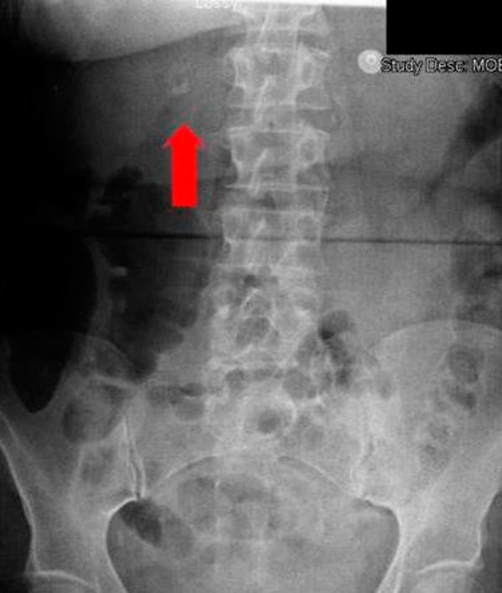
Plain erect chest radiograph, on close inspection a ring calcification can be seen in the right upper quadrant.

**Figure 2.**
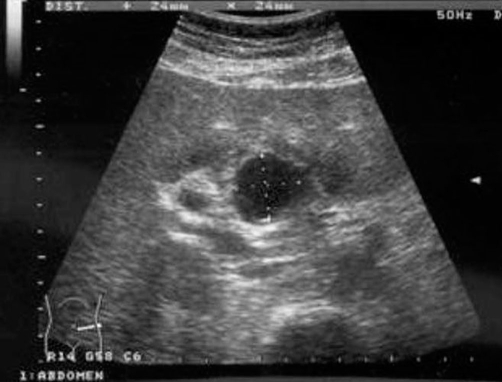
Ultrasound scan showing aneurysmal sac to the left of the common bile duct and portal vein.

A 62-year-old British Caucasian woman presented to the emergency department with worsening epigastric and right upper quadrant pain. Relevant medical history included diverticular disease and open cholecystectomy 15 years ago. On examination she was haemodynamically stable, abdominal examination revealed tenderness in the epigastrium and right hypochondrium. Blood tests showed; a C reactive protein of 297 mg/L, a white cell count of 12 ×10^9^/L, alkaline phosphatase of 137 IU/L, gamma glutamyl transpeptidase of 176 IU/L and other results including haemoglobin within normal ranges. Erect chest radiograph revealed a calcified ring in the Right Upper Quadrant (Figure 1). An ultrasound scan (USS) demonstrated arterial flow within a 24 mm × 24 mm cystic structure in relation to the common bile duct (CBD) and portal vein (PV) (Figure 2).

**Figure 3.**
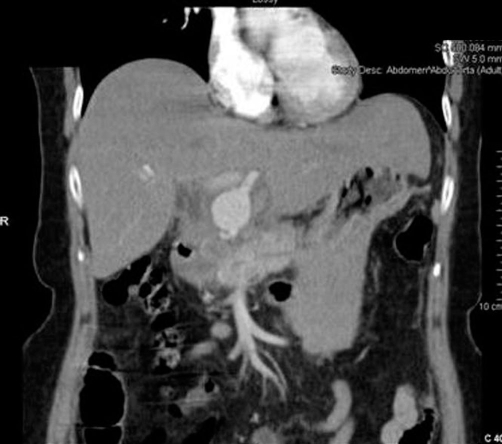
Computed tomography demonstrating left hepatic artery aneurysm.

**Figure 4.**
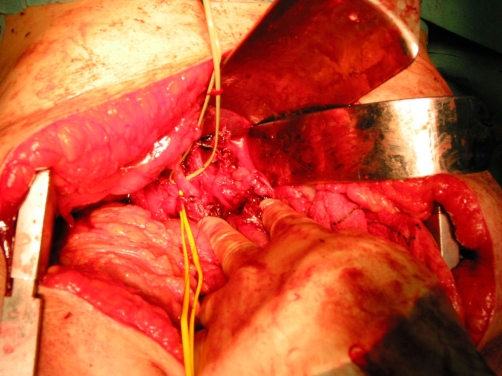
Intra-operative photograph showing isolation of common bile duct, portal vein and opened aneurysmal sac.

Urgent CT scan the following day demonstrated a 25 mm × 25 mm aneurysm of the left hepatic artery with minimal extravasation of contrast into the abdominal cavity. The CBD was dilated at 15mm with no intraductal calculi (Figure 3). Later that day she developed further severe abdominal pain and became haemodynamically unstable. In view of the sudden deterioration, referral for angiographic embolisation or stenting was not possible and an emergency exploratory laparotomy was performed. A midline laparotomy revealed 300 ml of haemoperitoneum with surrounding clots. Gentle dissection revealed a 25 mm ruptured true aneurymal sac (Figure 4) of the left hepatic artery. The proximal and distal parts of the artery were ligated with 2-0 vicryl from within the aneurysmal sac. The two flaps of the sac were then approximated with interrupted 2-0 vicryl. The patient was discharged the following week and followed up at 3 months with no evidence of liver dysfunction.

## Discussion

Hepatic artery aneurysms (HAAs) include aneurysms of the common, proper, left and right hepatic arteries. First described by Wilson in 1809 they constitute 20% of all visceral artery aneurysms [1-3]. Estimated incidences vary between 0.002% [2], and 0.4% [1]. Increasing number of cases are being diagnosed and commonly attributed to increased awareness, biliary interventions and better diagnostic imaging [1-3].

The natural history of HAAs are not well documented, however it is assumed that like any aneurysm can enlarge and rupture with life-threatening haemorrhage. Known aetiologies include atherosclerosis, mediointimal degeneration, trauma and infection (uncommon since the advent of antibiotics) [3,4]. A significant number are iatrogenic; following biliary procedures or cholecystectomy [3]. In our patient it is unlikely a previous cholecystectomy was associated with the HAA given the long time interval and that it was a true aneurysm and not the pseudoaneurysm associated with iatrogenic trauma [3].

Patients may present symptomatically or incidentally with findings on routine imaging. The most common symptom is epigastric or right upper quadrant pain as seen in our patient. Less than a third of patients will present with Quinke’s triad; abdominal pain, jaundice and haemobilia [1,3,4]. Incidental findings are sometimes apparent on abdominal films as rim calcifications [3,4]. Ultrasound scan may locate the aneurysm and demonstrate blood flow within it. CT scans are useful in demonstrating the nature of the aneurysm, adjacent structures and evidence of rupture [4]. However, angiography is considered gold standard as it provides the definitive diagnosis with size, shape and location of the aneurysm with scope for intervention [1,3,4].

Percutaneous embolisation is the most commonly used technique for treating HAAs. It is of particular value in intrahepatic aneurysms and high risk patients because it limits hepatic devascularization and is associated with lower morbidity [3]. Overall success rates are reported to be between 70 and 100% [1-3]. Regular follow up is recommended as re-canalisation may occur and repeat embolization necessary [3].

Surgical treatment consists of ligation or revascularization of the hepatic artery. The first successful ligation was performed by Kehr in 1903 [1,3,4]. Aneurysms of the common hepatic artery may be safely ligated provided there is a patent gastroduodenal artery or pancreaticoduodenal artery providing collateral blood supply to the liver [1-4]. If not there is a theoretical risk of liver ischaemia, this may be tested intra-operatively by occluding the artery in question and observing for cyanosis [1,3]. Aneurysms distal to the common hepatic artery should be treated by revascularization [1,3,4], however ligation of branch aneurysms are reported to be well tolerated [1]. In cases involving the right hepatic artery there is a possibility of gallbladder ischaemia and some authors recommend a simultaneous cholecystectomy [1].

## Conclusion

HAAs are rare but may present incidentally or as a cause of severe abdominal pain. Prompt diagnosis is paramount and crucial information may be gleamed from investigations performed in the emergency department such as plain abdominal radiographs and ultrasound scans. If a ruptured aneurysm is diagnosed, we recommend prompt referral to a surgical team for definitive management.
